# Supplementary material for: Impact of altitude on COVID-19 infection and death in the United States: A modeling and observational study
Source: PLoS One. 2021 Jan 14;16(1):e0245055. doi: 10.1371/journal.pone.0245055 (PMC7808593; doi:10.1371/journal.pone.0245055)

**Supplementary Figure 1.** Unprojected choropleth map of U.S. county centroids colored by elevation in meters. Elevation patterns based on county centroids closely represent elevation patterns of the continental U.S.


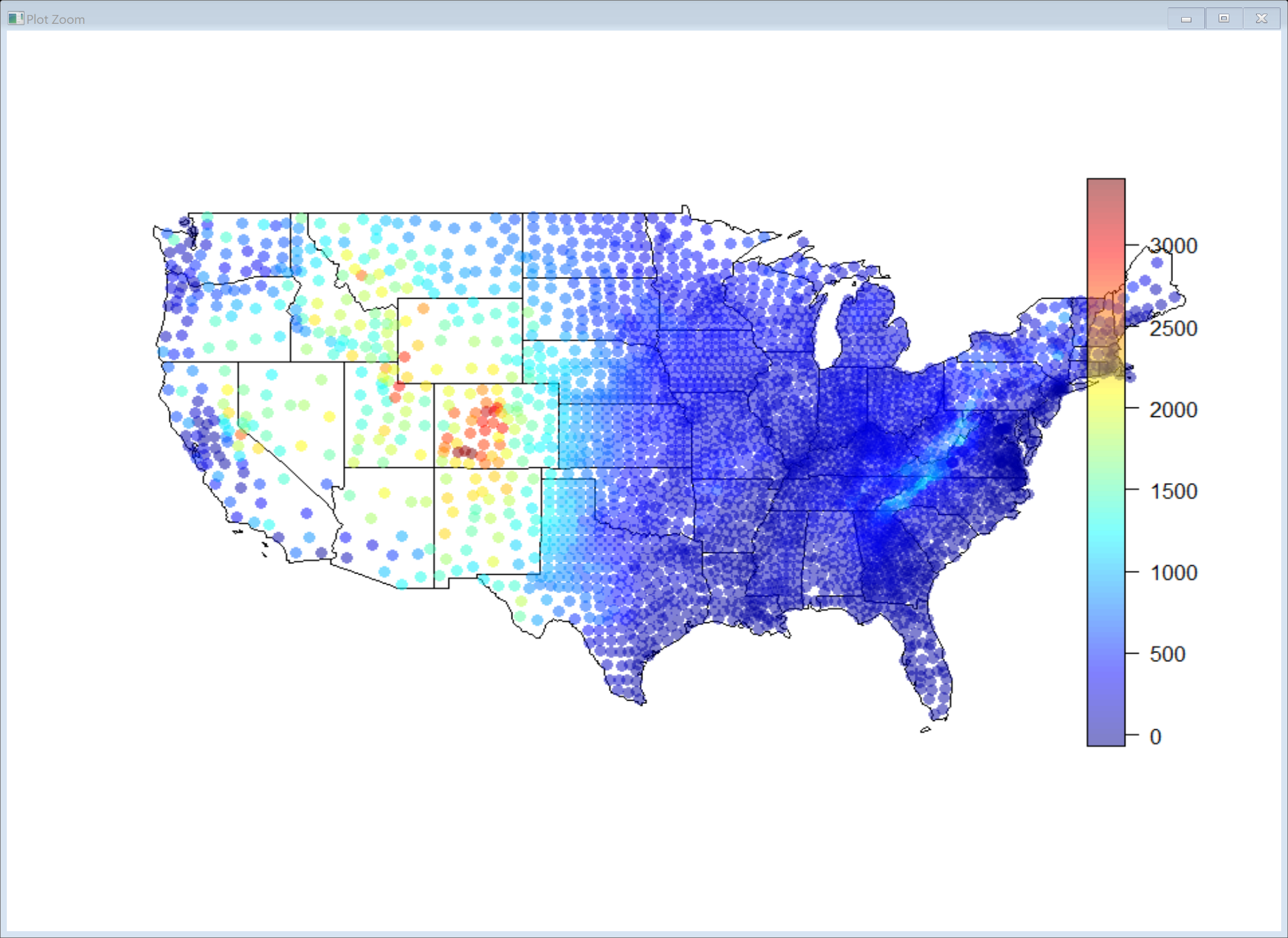

Supplement: S1 Fig — Elevation patterns based on county centroids closely represent elevation patterns of the continental U.S. (DOCX) [file pone.0245055.s001.docx]
