# Supplementary material for: Impact of altitude on COVID-19 infection and death in the United States: A modeling and observational study
Source: PLoS One. 2021 Jan 14;16(1):e0245055. doi: 10.1371/journal.pone.0245055 (PMC7808593; doi:10.1371/journal.pone.0245055)

**Supplementary Figure 2.** COVID-19 infection and death in matched high and low altitude counties with removal of counties with infection and death counts of zero. A) Mean COVID-19 cumulative per capita incidence per 100,000 population. B) Mean COVID-19 cumulative per capita death per 100,000 population. C) COVID-19 case mortality in high and low altitude counties of similar population density. N=33 for high altitude and N= 26 for low altitude counties. *p<0.05 by one-sided t-test.


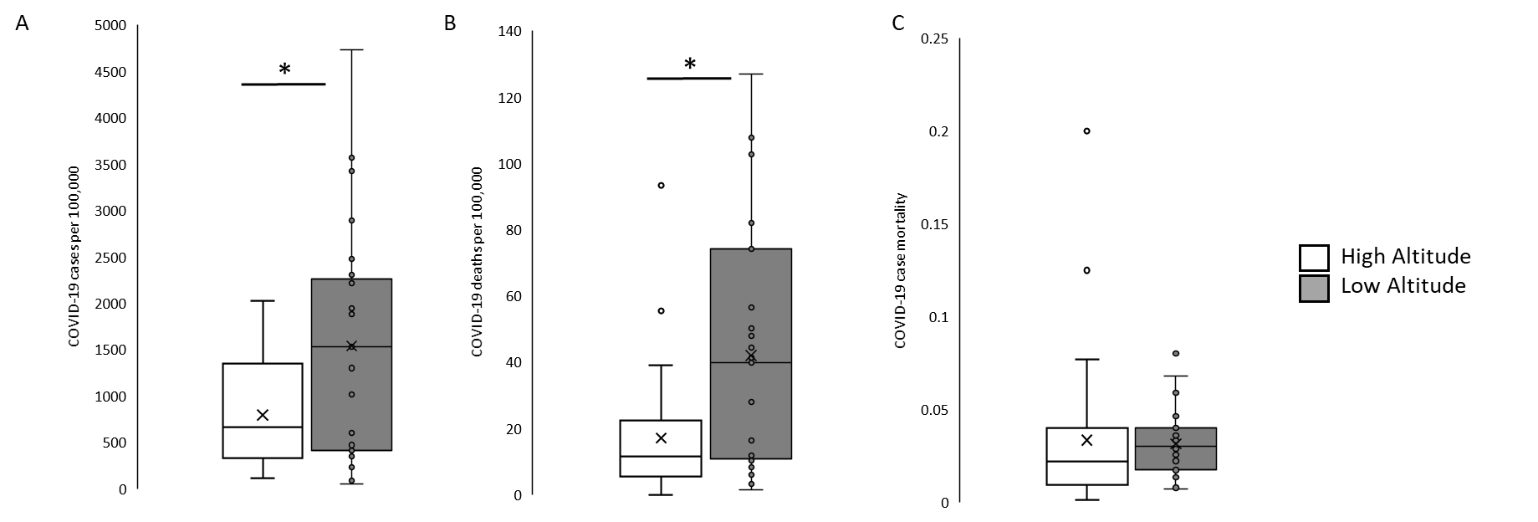

Supplement: S2 Fig — A) Mean COVID-19 cumulative per capita incidence per 100,000 population. B) Mean COVID-19 cumulative per capita death per 100,000 population. C) COVID-19 case mortality in high and low altitude counties of similar population density. N = 33 for high altitude and N = 26 for low altitude counties. *p<0.05 by one-sided t-test. (DOCX) [file pone.0245055.s002.docx]
