# Supplementary material for: Impact of altitude on COVID-19 infection and death in the United States: A modeling and observational study
Source: PLoS One. 2021 Jan 14;16(1):e0245055. doi: 10.1371/journal.pone.0245055 (PMC7808593; doi:10.1371/journal.pone.0245055)
Supplement: S1 Table — Smaller AIC and larger percent deviance explained constitutes the preferred model. (DOCX) [file pone.0245055.s003.docx]

**Supplementary Table 1.** Akaike Information Criterion (AIC) and percent deviance explained in parentheses for the statistical models considered. Smaller AIC and larger percent deviance explained constitutes the preferred model.

| Model**l** | **120-day incidence** | **90-day incidence** | **30-day incidence** |
| --- | --- | --- | --- |
| Tweedie-Poisson linear | 38886.60 (80.7%) | 37684.15 (84.3%) | **32932.59** (79.9%) |
| Tweedie-Poisson non-linear | **38881.62** (80.7%) | **37678.32** (84.2%) | 32933.56 (79.9%) |
| Neg-Binomial linear | 39361.39 (75.2%) | 38194.00 (77.5%) | 33501.09 (70.8%) |
| Neg-Binomial non-linear | 39343.09 (75.2%) | 38174.75 (77.5%) | 33497.49 (70.8%) |
