# Supplementary material for: Impact of altitude on COVID-19 infection and death in the United States: A modeling and observational study
Source: PLoS One. 2021 Jan 14;16(1):e0245055. doi: 10.1371/journal.pone.0245055 (PMC7808593; doi:10.1371/journal.pone.0245055)
Supplement: S1 File — (DOCX) [file pone.0245055.s004.docx]

**Supplementary Material**

**Statistical model specification**

Let $Y_{ij}$ be the number of incident COVID-19 cases in the *i*th US county and *j*th US state, and let $O_{i}$ be the number of persons at risk in that county, which was taken as the population at the time of the decennial census. Additionally, let $D(\ldots)$ be one of either the Negative Binomial or the Tweedie-Poisson distribution functions. In the case of the Negative Binomial, the customary dispersion parameter ($\kappa$) inside the variance function $\mu_{i}+\frac{\mu_{i}^{2}}{\kappa}$ was estimated. In the case of the Tweedie-Poisson, the power parameter (*p*) and the scale parameter were estimated. Collecting the distribution-specific parameters into the vector $\theta$, the regression model for $Y_{ij}$ was specified as follows:

$$Y_{ij}\sim D(\mu_{ij},\theta)$$

$$\log\left( \mu_{ij} \right)=\log\left( O_{i} \right)+\beta_{0}+\boldsymbol{x}_{i}^{'}\boldsymbol{\beta}+u_{i}+v_{j}$$

$$u_{i}\sim ICAR(\mathbf{W})$$

$v_{j}\sim N(0,\sigma_{v}^{2})$.

In the equation above, $\beta_{0}$ is the intercept term, $\boldsymbol{x}_{i}^{'}$ contains covariate values of the *i*th county, including the rural-urban continuum codes, number of persons per household, the interaction of rural-urban continuum codes and the number of persons per household, and county centroid elevation in meters. County-specific latent spatial effects $u_{i}$ were specified using the Markov Random Field (MRF) smooth basis function, or equivalently, the Intrinsic Conditionally Auto-Regressive (ICAR) adjacency-based model (e.g., Paciorek, 2013). The matrix **W** is the county adjacency matrix that was specified using “queen” adjacency rules. State-specific latent effects $v_{j}$ are mutually independent and therefore constitute random intercepts. All quantitative variables were centered and scaled prior to analysis. Estimation took place using the gam() function in the mgcv R package (e.g., Wood, 2011) using the REML smoothing parameter estimation method.

**Statistical model selection**

Both the Negative-Binomial and the Tweedie-Poisson families were considered in this modeling exercise. The latter is known to be more flexible than the former in the presence of excess zero counts and/or heavy tails (e.g., El-Shaarawi et al., 2011). In addition to the distribution function, we also considered both linear and non-linear functions of elevation. The non-linear function of elevation was specified using the Duchon basis to avoid edge effects. For case counts based on all three of 30, 90, and 120 days, the Tweedie-Poisson family had lower AIC and higher percent deviance explained, which is akin to the unadjusted R-squared (Supplementary Table 1). Non-linear association between elevation and incidence was preferred for 120-day and 90-day incidence, but not 30-day incidence, where the association was essentially linear. Finally, all models were deemed to fit the data well with percent deviance explained of 80.7%, 84.2%, and 79.9% for 120-day, 90-day, and 30-day incidence, respectively.

**Supplementary References**

El‐Shaarawi, A.H., Zhu, R. and Joe, H. (2011), Modelling species abundance using the Poisson–Tweedie family. Environmetrics, 22: 152-164. doi:10.1002/env.1036

Paciorek, Christopher J. Spatial models for point and areal data using Markov random fields on a fine grid. Electron. J. Statist. 7 (2013), 946--972. doi:10.1214/13-EJS791. https://projecteuclid.org/euclid.ejs/1366031046

Wood, S.N. (2011) Fast stable restricted maximum likelihood and marginal likelihood estimation of semiparametric generalized linear models. Journal of the Royal Statistical Society (B) 73(1):3-36
